# Prognostic nutritional index is an independent prognostic factor for older patients aged ≥ 85 years treated by gastric endoscopic submucosal dissection

**DOI:** 10.1186/s12876-021-01896-1

**Published:** 2021-08-23

**Authors:** Yosuke Toya, Masaki Endo, Risaburo Akasaka, Toshifumi Morishita, Shunichi Yanai, Shotaro Nakamura, Makoto Eizuka, Ryo Sugimoto, Noriyuki Uesugi, Tamotsu Sugai, Takayuki Matsumoto

**Affiliations:** 1 Division of Gastroenterology, Department of Internal Medicine, School of Medicine, Iwate Medical University, Idaidori 1-1-1, Yahaba, 028-3694 Japan; 2 Kaiunbashi Endoscopy Clinic, Morioka, Japan; 3 Division of Molecular Diagnostic Pathology, Department of Pathology, School of Medicine, Iwate Medical University, Yahaba, Japan

**Keywords:** Gastric cancer, Endoscopic submucosal dissection, Older patients, Prognostic factors, Prognostic nutritional index

## Abstract

**Background:**

Clinical outcomes and prognostic factors for survival after endoscopic submucosal dissection (ESD) in older patients aged ≥ 85 years with early gastric cancer (EGC) are not well defined. The aim of this study was to investigate the clinical outcomes and prognostic factors for survival after ESD in older patients aged ≥ 85 years with EGC.

**Methods:**

Clinical outcomes of 70 patients aged ≥ 85 years with EGC treated with ESD were evaluated retrospectively. Prognostic factors for overall survival (OS) were analyzed with the Kaplan–Meier method and a Cox proportional hazards model.

**Results:**

During the follow-up period, 33 patients died from any cause, none of whom died from gastric cancer. OS probability after 3 years was 90.0%. Univariate analyses revealed that a neutrophil/lymphocyte ratio ≥ 2.6, a prognostic nutritional index (PNI) < 42.5 and low serum albumin value (< 3.5 g/dl) were associated with poor OS. Cox multivariate analysis revealed low PNI (< 42.5) to be an independent prognostic factor associated with OS (hazard ratio; 3.40, 95% confidence interval; 1.47–7.86, *P* = 0.004).

**Conclusions:**

PNI may be a useful parameter for making the decision to perform ESD for older patients aged ≥ 85 years with EGC.

## Background

Endoscopic submucosal dissection (ESD) has been widely accepted as an established treatment for early gastric cancer (EGC) with a negligible risk of lymph node metastasis [1–5]. Recently, several studies have revealed excellent short- and long-term outcomes after ESD for EGC, even in patients with non-curative ESD and in older patients with EGC [6–15].

In recent decades, the older population has been increasing rapidly worldwide. Gastric cancer is still an important cause of death in Japan. Due to the increasing necessity of ESD for older patients, however, physicians are facing a problem as to the indications for gastric ESD in older patients with multiple comorbidities. To date, several studies have found various prognostic factors for survival in patients undergoing ESD for EGC [12–15]. However, few studies have identified prognostic factors in patients with EGC aged 85 years or older who underwent ESD [9, 12]. Clarifying the prognostic factors in super-elderly patients is thus needed to establish the indications for gastric ESD in this population. The aim of this study was to investigate the clinical outcomes and prognostic factors for survival after ESD in older patients aged ≥ 85 years with EGC.

## Methods

### Patients

**Fig. 1:**
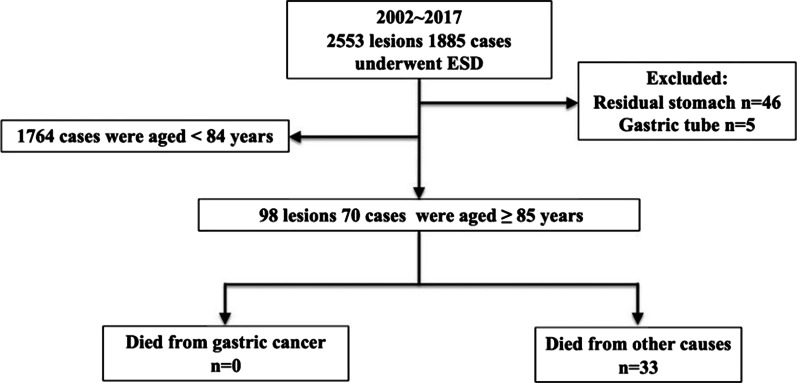
Flow of patients enrolled in the study

We performed ESD for 1,885 patients with 2,553 EGCs at our institute during the period from June 2002 to December 2017. Among those, 46 patients with prior gastric surgery and 5 patients with EGC in the gastric tube were excluded. Of the remaining 1,834 patients, we recruited 70 patients aged ≥ 85 years for the present study (Fig. 1).

Patients’ medical charts at the time of gastric ESD were verified to obtain data on clinical and demographic characteristics, including age, gender, Eastern Cooperative Oncology Group (ECOG) performance status (PS) [16], and body mass index (BMI). We evaluated the following items as possible prognostic factors: Geriatric Nutritional Risk Index (GNRI) [17, 18], Charlson comorbidity index (CCI) [19], neutrophil to lymphocyte ratio (NLR) [20] and prognostic nutritional index (PNI) [21]. The GNRI, CCI, NLR and PNI were calculated with the methods that we reported previously [13].

This study was performed following the Helsinki Declaration of the World Medical Association. Written informed consent was obtained from all patients before ESD. Informed consent for study enrollment was obtained in the form of an opt-out on the website. This study protocol was approved by the ethical committee of Iwate Medical University (MH2020-169).

### Curability criteria

Curability of ESD was determined based on guidelines reported by the Japanese Gastric Cancer Association (JGCA) [22] and the Japanese Gastroenterological Endoscopy Society (JGES) [23]. When a lesion was resected *en bloc*, was predominantly a differentiated type, pathologically intramucosal carcinoma (pT1a) and was free from lymphovascular invasion (ly0, v0) with negative surgical margins (R0), the procedure was classified as endoscopic curability (eCura) A. When a lesion was resected *en bloc* and it was (1) ≤ 2 cm, predominantly an undifferentiated type, pT1a, and UL (−) with negative surgical margins (R0); or (2) ≤ 3 cm, predominantly a differentiated type, pathologically minute submucosal (SM) cancer < 500 μm (pT1b/SM1) in vertical depth with negative surgical margins (R0), the procedure was classified as eCuraB. The resection was classified as endoscopic curability eCuraC when the resected specimen did not fulfill the conditions of eCuraA or eCuraB. The resection was regarded as eCuraC-1 when the lesion was a histologically differentiated type and fulfilled the other criteria to be classified as either eCuraA or eCuraB, but was either not resected *en bloc* or had a positive horizontal margin. All other eCuraC resections were subclassified as eCuraC-2.

### Follow-up and collection of outcomes data

In principle, we followed the subjects according to eCura status. For patients of eCuraA status, an endoscopic examination was conducted once a year. For patients of eCuraB status, an endoscopic examination was conducted once a year, and computed tomography (CT) was also performed once a year. The decision to carry out either additional gastrectomy or follow-up without gastrectomy was determined by the attending physician for each patient of eCuraC-2 status, taking into consideration the risk of gastrectomy. For patients who were followed without gastrectomy, an endoscopic examination was conducted 1 to 3 months after ESD. Thereafter, endoscopic examinations were conducted 6 and 12 months after ESD. Unless local recurrence was found, we continued subsequent annual endoscopic examinations. Abdominal ultrasound and CT were also performed once a year.

If any local recurrence was found, the attending endoscopists discussed the indication for additional ESD. However, the final decision regarding additional treatment or follow-up without treatment was made by the patient after discussion with the attending physician. *Helicobacter pylori* (*H. pylori*) was eradicated in infected patients immediately after ESD. For patients who were followed up outside of our institution, we conducted an annual questionnaire survey via their primary care physicians. For patients who did not visit regularly, we contacted them or their family members directly to confirm the prognosis.

### Statistical analysis

Overall survival (OS) after ESD was analyzed with the Kaplan–Meier method, and differences between groups were assessed with the log-rank test. The relationship between OS and each clinicopathologic factor was analyzed by univariate analysis with the log-rank test. Cut-off values for the GNRI were determined based on a previous report [13]. Cut-off values for the NLR, the PNI and the serum albumin value were determined by receiver operating characteristic (ROC) analysis. Values that maximized the sensitivity and specificity for OS were used as the cut-off values. Multivariate analyses were performed using a Cox proportional hazards regression model with stepwise selection method. In each analysis, a *P* value < 0.05 was considered statistically significant. All statistical analyses were performed with SPSS version 25 software for MAC OS (SPSS Inc., Chicago, IL, USA) and JMP version 14 (Statistical Discovery Program, Cary, NC, United States).

## Results

Table 1 shows the demographic and clinical characteristics of the study population. The median age was 86 years, with a predominance of males (60.0%). The ECOG PS was 0 or 1 in 61 patients (87.2%). The median BMI was 22.6 kg/m^2^, and the median follow-up period was 6.0 years. The mean GNRI was 101.8, and 59 patients (84.3%) had a CCI of 0–2. The median NLR was 2.4, and the mean PNI was 47.2.

**Table 1:** Demographic and clinical characteristics of 70 patients aged ≥ 85 years who underwent ESD for gastric cancer

Age, years, median (range, IQR)	86 (85–92, 2.0)
Gender, n (%)	
Male	42 (60.0)
Female	28 (40.0)
ECOG PS, n (%)	
0	41 (58.6)
1	20 (28.6)
2	9 (12.9)
Body mass index, kg/m^2^, median (range, IQR)	22.6 (16.2–33.6, 4.0)
Follow-up period, years, median (range, IQR)	6.0 (0.33–13.9, 4.2)
GNRI, mean (± SD)	101.8 (± 9.0)
CCI, n (%)	
0	30 (42.9)
1	22 (31.4)
2	7 (10.0)
3	6 (8.6)
4	2 (2.9)
5	2 (2.9)
6	1 (1.4)
NLR, median (range, IQR)	2.4 (0.7–10.4, 1.3)
PNI, mean (± SD)	47.2 (± 4.9)

ESD, endoscopic submucosal dissection; IQR, interquartile range; ECOG PS, Eastern Cooperative Oncology Group performance status; SD, standard deviation; GNRI, geriatric nutritional risk index; CCI, Charlson comorbidity index; NLR, neutrophil to lymphocyte ratio; PNI, prognostic nutritional index

The clinicopathological characteristics of the resected EGCs are summarized in Table 2. Of the 70 tumors, 64 (91.4%) were initial lesions, and 6 (8.6%) were metachronous lesions. The most frequent location was the lower third of the stomach, and the median tumor size was 13 mm. Most tumors exhibited a histologically differentiated type (98.6%), and 3 tumors (4.3%) had invaded the deep portion of the submucosa. Lymphatic invasion was positive in 4 tumors (5.7%), while vascular invasion was not found. Ulcerative findings were identified in 7 tumors (10.0%). There were 58 patients with EGC of eCuraA status (82.9%), 4 patients with EGC of eCuraB status (5.7%) and 8 patients with EGC of eCuraC-2 status (11.4%). All patients of eCuraC-2 status were followed up without additional treatment. The median procedure time for ESD was 37.5 min. With regard to adverse events, postoperative bleeding and perforation each occurred in one patient.

**Table 2:** Clinicopathological characteristics of 70 patients aged ≥ 85 years who underwent ESD for gastric cancer

Tumor type, n (%)	
Initial lesion	64 (91.4)
Metachronous lesion	6 (8.6)
Tumor location, n (%)	
Upper	13 (18.6)
Middle	23 (32.9)
Lower	34 (48.6)
Tumor size, mm, median (range, IQR)	13.0 (4–95, 9.0)
Macroscopic appearance, n (%)	
Elevated	40 (57.1)
Depressed/flat	30 (42.9)
Histology, n (%)	
Differentiated type	69 (98.6)
Undifferentiated type	1 (1.4)
Depth, n (%)	
M/SM1	67 (95.7)
SM2	3 (4.3)
Lymphatic invasion, n (%)	4 (5.7)
Vascular invasion, n (%)	0 (0)
Ulcerative findings, n (%)	7 (10.0)
Curability n (%)	
eCuraA	58 (82.9)
eCuraB	4 (5.7)
eCuraC-2	8 (11.4)
Additional gastrectomy, n (%)	0 (0)
Procedure time, min, median (range, IQR)	37.5 (6–415, 41)
Postoperative bleeding, n (%)	1 (1.4)
Perforation, n (%)	1 (1.4)
Other metachronous cancers, n (%)	0 (0)
Death due to any causes, n (%)	33 (47.1)
Death due to gastric cancer, n (%)	0 (0)

ESD, endoscopic submucosal dissection; IQR, interquartile range; M, mucosa; SM1, superficial portion of the submucosa within 500 μm from the muscularis mucosae; SM2, deep portion of the submucosa ≥ 500 μm from the muscularis mucosae; min, minutes

During the follow-up period (6.0 years, median), recurrence of primary EGC was noted in a patient of eCuraC-2 status. The primary EGC of the patient invaded the superficial portion of the submucosa within 500 μm from the muscularis mucosae (SM1) with positive lymphatic invasion and negative resected margins. The patient was followed up without any additional treatment and died of aspiration pneumonia three and a half year after ESD. In the remaining 69 patients, neither recurrent nor metachronous GC was found during the follow-up period.

**Fig. 2:**
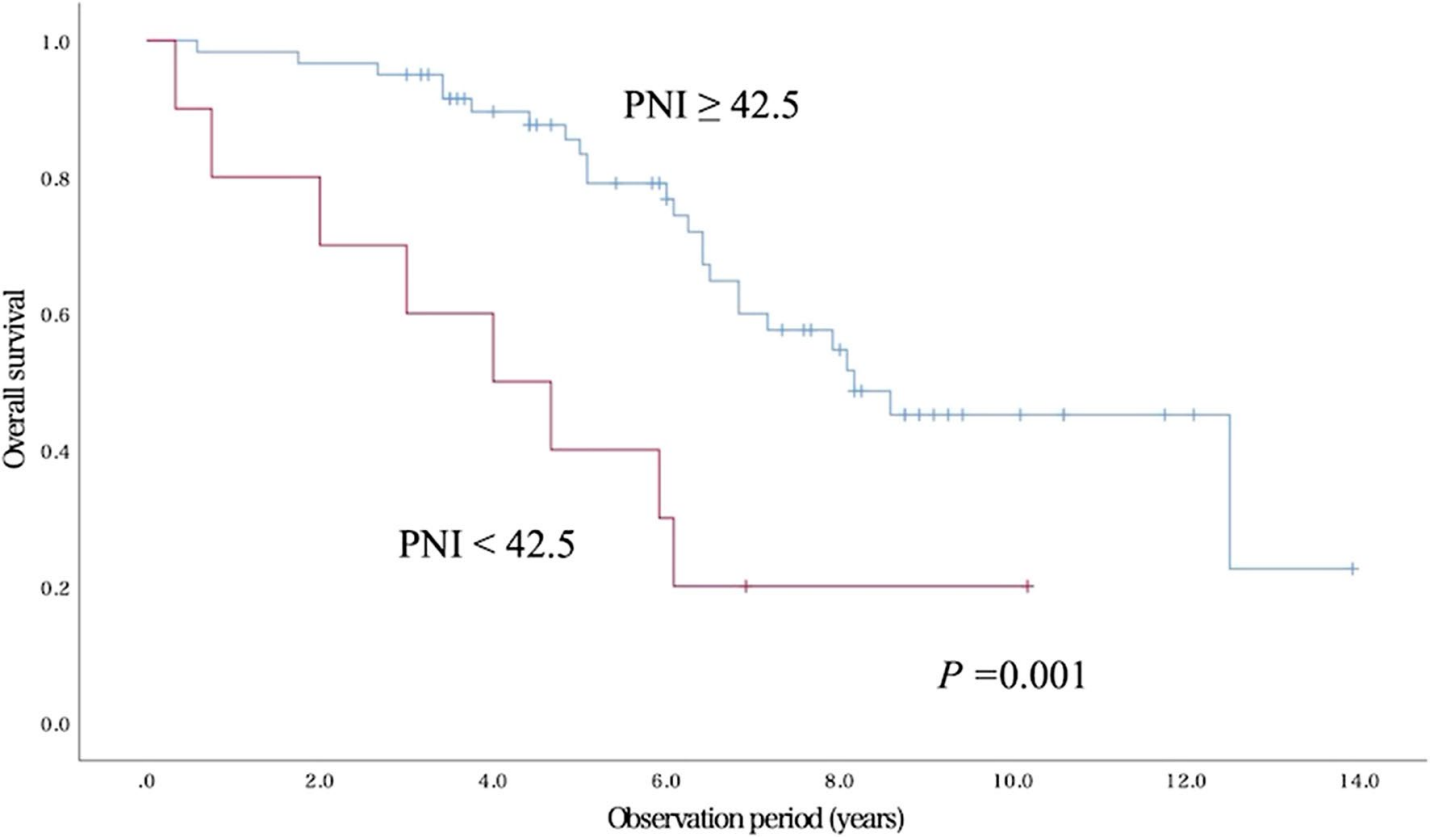
Overall survival curves for patients in the low PNI group and the high PNI group (*P* = 0.001)

During the follow-up period, 33 patients died from any cause, but no patient died from GC. The most common cause of death was cardiovascular disease in 11 patients, followed by pneumonia in 9 patients and senility in 7 patients. Probability of OS after 3 years was 90.0%. Results of univariate analyses for possible prognostic factors are summarized in Table 3. Patients who had a high NLR (≥ 2.6), a low PNI (< 42.5) and a low albumin value (< 3.5 g/dl) were found to have a lower OS than the other patients. As shown in Table 4, a Cox proportional hazards model indicated that only low PNI (< 42.5) was an independent prognostic factor associated with OS (hazard ratio, 3.40; 95% CI, 1.47–7.86; *P* = 0.004). The overall survival rate was significantly lower in the low PNI group than in the high PNI group (Fig. 2, *P* = 0.001).

**Table 3:** Overall survival by the Kaplan–Meier method

Variable	No. of patients	OS	*P* value
Gender			
Male	42	0.50	0.19
Female	28	0.57	
ECOG PS			
0–1	61	0.57	0.11
2–3	9	0.22	
GNRI			
≥ 92	60	0.55	0.35
< 92	10	0.40	
CCI			
0–2	59	0.54	0.22
≥ 3	11	0.46	
NLR			
≥ 2.6	30	0.43	0.066
< 2.6	40	0.60	
PNI			
≥ 42.5	60	0.58	0.001
< 42.5	10	0.20	
Serum albumin			
≥ 3.5 g/dl	60	0.58	0.029
< 3.5 g/dl	10	0.20	
Tumor location			
Upper	13	0.62	0.98
Middle	23	0.48	
Lower	34	0.53	
Tumor size			
≥ 20 mm	17	0.53	0.66
< 20 mm	53	0.53	
Macroscopic appearance			
Elevated	40	0.45	0.11
Depressed/flat	30	0.63	
Histology			
Differentiated type	69	0.52	0.60
Undifferentiated type	1	1.00	
Depth of invasion			
M/SM1	67	0.51	0.63
SM2	3	1.00	
Lymphatic invasion			
Present	4	0.50	0.88
Absent	66	0.53	
Ulcerative findings			
Present	7	0.43	0.80
Absent	63	0.54	
Curability			
eCuraA/B	62	0.53	0.76
eCuraC-2	8	0.50	

OS, overall survival; ECOG PS, Eastern Cooperative Oncology Group performance status; GNRI, geriatric nutritional risk index; CCI, Charlson comorbidity index; NLR, Neutrophil to lymphocyte ratio; PNI, Prognostic nutritional index; M, mucosa; SM1, superficial portion of the submucosa within 500 μm from the muscularis mucosae; SM2, deep portion of the submucosa ≥ 500 μm from the muscularis mucosae

**Table 4:** Results of multivariate analysis for factors associated with overall survival

Variable	HR	95% CI	*P* value
NLR ≥ 2.6	1.91	0.93–3.90	0.078
PNI < 42.5	3.40	1.47–7.86	0.004
ECOG PS ≥ 2	2.25	0.92–5.50	0.074

HR, hazard ratio; CI, confidence interval; NLR, Neutrophil to lymphocyte ratio; PNI, Prognostic nutritional index; ECOG PS, Eastern Cooperative Oncology Group performance status

## Discussion

In the present study, we clarified the clinical outcomes and prognostic factors for survival in older patients aged ≥ 85 years with EGC treated with gastric ESD. Among 70 patients, 33 patients died, but no patient died from GC during the follow-up period. Among various prognostic indices, multivariate analysis revealed low PNI (< 42.5) to be an independent prognostic factor for survival after ESD. It was also suggested that among the items included in PNI, serum albumin was the significant indicator for prognosis. To our knowledge, this is the longest follow-up period study to report the long-term outcomes and prognostic factors of older patients with EGC after ESD.

Recently, a new system for the determination of the feasibility of gastric ESD has been proposed by the Japanese Gastric Cancer Association (JGCA) [22] and the Japanese Gastroenterological Endoscopy Society (JGES) [23]. eCuraA status corresponds to conventional curative resection, eCuraB status corresponds to expanded curative resection and eCuraC status corresponds to non-curative resection. Although sufficient long-term outcomes after gastric ESD have not yet been accumulated, patients of eCuraB status are presumed to be cured by gastric ESD. In fact, we found a local recurrence of GC in a patient of eCuraC2 status among our older population.

PNI is an index proposed by Onodera et al. for assessing surgical risk in patients with advanced gastrointestinal cancer [21]. PNI is calculated with the serum albumin level and the total lymphocyte count. The index has been widely used as a nutritional index because of its simplicity and high reliability. It has been reported that PNI was closely associated with prognosis of several cancers, including GC [24, 25], hepatocellular carcinoma [26, 27], and pancreatic cancer [28]. Also, subjects with low PNI are shown to be at the risk of high mortality in acute heart failure [29, 30]. More recently, it has been reported that PNI was an appropriate predictor of severity of the coronavirus disease 2019 (COVID-19) [31].

Iwai et al. reported that CCI and PNI were prognostic indicators for non-elderly and elderly patients with EGC treated with ESD [14]. They classified their study subjects into two groups, elderly (≥ 80 years) or non-elderly (< 80 years) and showed that OS among patients with a low CCI (≤ 2) and a high PNI (≥ 47.7) was significantly higher than in patients with high CCI (≥ 3) and a low PNI (< 47.7), regardless of age. Sekiguchi et al. also reported that a low PNI was a prognostic factor in patients aged > 85 years with EGC treated with ESD, showing that OS was significantly lower in patients with a low PNI (< 44.6) than in patients with a high PNI (≥ 44.6) [12]. Although the age of the subjects varied, and the cut-off value of PNI was not the same in those studies, the trends in the association of prognostic factors and OS are similar to those found in our present study. We thus believe that PNI is an important predictor of prognosis in older patients with EGC.

We previously reported that a CCI ≥ 3 was an independent prognostic factor for survival in older patients aged ≥ 75 years with EGC after non-curative ESD [13]. In contrast, the CCI was not found to be a prognostic factor, even on univariate analysis, in our older patients aged ≥ 85 years in the current study. While the CCI is calculated with past and present comorbidities, the PNI is calculated with laboratory test results, including real-time nutritional status. We thus consider that the PNI may be more useful for the prediction of prognosis in a shorter period than the CCI.

In this study, no patient died of GC after ESD among all patients with EGC. In 2019, the average life expectancy in Japan was estimated to be 81.41 years for males and 87.45 years for females [32]. Tsukuma et al. calculated the median time from the initial diagnosis of early GC to advanced cancer to be 44 months [33]. In our subjects, the median time from ESD to death in the low PNI (< 42.5) group was 41 months. This observation may suggest that patients aged ≥ 85 years with a low PNI have a life expectancy less than the median time until the development of advanced cancer. We thus believe that careful follow-up without ESD may be an acceptable option for older patients aged ≥ 85 years with EGC with a provisional poor prognosis by PNI. In clinical practice, PNI can be an objective indicator in shared decision making for the treatment of GC in the older patients. For older patients with a high PNI, either ESD or surgery seem to be inevitable to prevent GC-related death. Further evaluation in a multicenter, prospective study is warranted to validate this speculation.

The present study has several limitations. First, the retrospective nature of the study introduced selection bias. In particular, we included patients who were selected by physicians, but patients with GC, who could not undergo ESD because of poor performance status, were excluded. Second, the sample size of the present study was small due to the single-institutional nature of the data collection. A prospective, multi-center study to compare OS between groups dichotomized by PNI is needed to validate our observation.

## Conclusions

Our study showed that a low PNI (< 42.5) was a single, independent prognostic factor associated with OS in older patients aged ≥ 85 years with EGC treated with ESD. Based on the results of this study, the PNI is suggested to be a factor for decision-making regarding gastric ESD in the older population.

## Data Availability

The datasets used and/or analyzed during the current study are available from the corresponding author on reasonable request.

## Abbreviations

ESD: Endoscopic submucosal dissection
EGC: Early gastric cancer
OS: Overall survival
NLR: Neutrophil/lymphocyte ratio
PNI: Prognostic nutritional index
ECOG: Eastern Cooperative Oncology Group
PS: Performance status
BMI: Body mass index
GNRI: Geriatric Nutritional Risk Index
eCura: Endoscopic curability
SM: Submucosal
CT: Computed tomography
ROC: Receiver operating characteristic
